# CRL3^IBTK^ Regulates the Tumor Suppressor Pdcd4 through Ubiquitylation Coupled to Proteasomal Degradation*

**DOI:** 10.1074/jbc.M114.634535

**Published:** 2015-04-16

**Authors:** Antonio Pisano, Simona Ceglia, Camillo Palmieri, Eleonora Vecchio, Giuseppe Fiume, Annamaria de Laurentiis, Selena Mimmi, Cristina Falcone, Enrico Iaccino, Annarita Scialdone, Marilena Pontoriero, Francesca Fasanella Masci, Rosanna Valea, Shibu Krishnan, Marco Gaspari, Giovanni Cuda, Giuseppe Scala, Ileana Quinto

**Affiliations:** From the Department of Experimental and Clinical Medicine, University “Magna Graecia” of Catanzaro, 88100 Catanzaro, Italy

**Keywords:** E3 ubiquitin ligase, serum, translation regulation, tumor suppressor gene, ubiquitylation (ubiquitination), IBtk, Pdcd4

## Abstract

The human inhibitor of Bruton's tyrosine kinase isoform α (IBtkα) is a BTB protein encoded by the *IBTK* gene, which maps to chromosomal locus 6q14.1, a mutational hot spot in lymphoproliferative disorders. Here, we demonstrate that IBtkα forms a CRL3^IBTK^ complex promoting its self-ubiquitylation. We identified the tumor suppressor Pdcd4 as IBtkα interactor and ubiquitylation substrate of CRL3^IBTK^ for proteasomal degradation. Serum-induced degradation of Pdcd4 required both IBtkα and Cul3, indicating that CRL3^IBTK^ regulated the Pdcd4 stability in serum signaling. By promoting Pdcd4 degradation, IBtkα counteracted the suppressive effect of Pdcd4 on translation of reporter luciferase mRNAs with stem-loop structured or unstructured 5′-UTR. IBtkα depletion by RNAi caused Pdcd4 accumulation and decreased the translation of Bcl-xL mRNA, a well known target of Pdcd4 repression. By characterizing CRL3^IBTK^ as a novel ubiquitin ligase, this study provides new insights into regulatory mechanisms of cellular pathways, such as the Pdcd4-dependent translation of mRNAs.

## Teaser

**Background:** IBtkα is an uncharacterized protein belonging to the family of BTB proteins.

**Results:** IBtkα is the substrate receptor for a Cullin3-dependent ubiquitin ligase promoting ubiquitylation and proteasomal degradation of Pdcd4.

**Conclusion:** By regulating Pdcd4 stability, IBtkα can modulate the translation of specific mRNAs under different cellular conditions.

**Significance:** The identification of new players in the ubiquitin/proteasome pathways contributes to a better understanding of protein homeostasis.

## Introduction

Protein ubiquitylation is an essential process for proteasome-mediated degradation or signalosome recruitment of proteins in response to extracellular stimuli (1, 2). Ubiquitylation occurs through three sequential steps, where ubiquitin (Ub)^5^ is first activated by the Ub-activation enzyme (E1) and then transferred to the Ub-conjugating enzyme (E2) and finally attached to the protein substrate by the Ub ligase (E3) (1). The largest family of E3 ligases consists of multisubunit complexes, including the scaffold proteins of the Cullin (Cul) family, which are named Cul-RING ligases (CRLs) (3). CRLs are composed of three major elements: (i) Cul protein; (ii) the catalytic module, composed of a RING finger protein (RBX1 or RBX2), which interacts with the C-terminal domain of Cul and recruits the Ub-conjugating enzyme (E2); (iii) the substrate recognition module, which interacts with the N-terminal domain of Cul and places the substrate in close proximity of the catalytic module, thus facilitating the Ub transfer (4). NEDD8 covalent modification of the C-terminal domain of Cul is additionally required to induce the conformational changes of CRL structure for bringing the substrate and E2-ubiquitin into juxtaposition (5).

The best characterized CRL is the Skp1/Cul1/F-Box (SCF) complex, where Cul1 binds to Rbx1 and to the adaptor protein Skp1, which in turn associates with the F-box protein responsible for substrate recruitment to the SCF complex (6–8). A similar molecular organization is observed in Cul3-based CRL (CRL3), which consists of Cul3, Rbx1, and the substrate-specific adaptor with one or more Bric-a-brac, Tramtrack, and Broad complex/Pox virus and zinc finger (BTB/POZ) domain (9, 10), hereafter referred to as the BTB domain. BTB proteins bind to Cul3 via the BTB domain (11) and determine the substrate specificity of the ubiquitin ligase complex through an additional protein-protein interaction domain, including the MATH (meprin and TRAF homology) domain, Kelch (KLH) repeats, zinc fingers, or ankyrins (12). Hence, the BTB proteins incorporate the features of the Skp1/F-box dimer of CRL1 within a single polypeptide (11, 13–15).

The BTB domain is evolutionarily conserved and mediates a variety of biological processes, such as transcriptional regulation, ion channel assembly, cytoskeleton dynamics, apoptosis, and protein ubiquitylation (3, 8). In *Schizosaccharomyces pombe*, three BTB proteins (Btb1, Btb2, and Btb3) have been identified, which function as substrate receptors of Pcu3, the yeast Cul3 orthologue (14). The human genome encodes nearly 200 BTB proteins (16), with only a small subset having been characterized as substrate adaptors of CRL3 (12). A common structural feature of BTB proteins of CRL3 is the presence of a paired helical structure, named the 3-box motif, which consists of a two-helix extension of the BTB domain that is critical for high affinity interaction with Cul3 (12, 17). A well known substrate receptor of CLR3 is the BTB/Kelch protein Keap1, which promotes the ubiquitylation coupled to proteasomal degradation of Nrf2, a transcriptional factor involved in oxidative stress response (18–21). Other substrates of mammalian Cul3 include Dishevelled of Wnt signaling, Aurora B kinase, cyclin E1, RhoA, WNK kinase isoforms, the GluR6 kainate receptor, Daxx, RhoBTB2, topoisomerase 1, and Ci (12). The relevance of BTB proteins in human pathologies has been highlighted by mutations of BTB proteins that are responsible for diseases, such as gigaxonin in giant axonal neuropathy (22), KLHL9 in autosomal dominant distal myopathy (23), autosomal dominant retinitis pigmentosa (22), and Gordon's hypertension syndrome (24). From this perspective, the functional characterization of novel BTB proteins is relevant for substrate specificity of CRL3 and their implication in human diseases.

The human inhibitor of Bruton's tyrosine kinase (*IBTK*) gene maps at the 6q14.1 cytogenetic location, which is a region of recurrent chromosomal aberrations in lymphoproliferative disorders (25). The *IBTK* gene has a complex organization because it expresses three coding transcripts for IBtkα, -β, and -γ protein isoforms and additional non-coding transcripts, including the pre-miRNA IBTK (26, 27). IBtkγ is the first identified 26-kDa protein isoform that acts as an inhibitor of Btk in B-cell receptor signaling (25, 28). IBtkα is the most highly and ubiquitously expressed protein isoform with a molecular mass of 150 kDa and has not been functionally characterized. IBtkα harbors multiple domains, including two ankyrin repeats at the N terminus, followed by three regulator of chromosome condensation 1 (RCC1) domains, two separated BTB domains, and a large C-terminal region of about 500 amino acid residues with no recognizable motifs (26). IBtkα is structurally related to *S. pombe* Btb1, a substrate receptor of the yeast Pcu3 (Cul3)-based ubiquitin ligase complex (11, 14). Based on the structural homology of IBtkα with Btb1, in this study, we addressed the question of whether IBtkα was a substrate receptor of CRL3-recruiting proteins for ubiquitylation and subsequent degradation by the proteasome.

## Experimental Procedures

### 

#### 

##### Plasmids, siRNAs, Lentiviruses, and Antibodies

pCMV6-IBtkα-FLAG (RC218657, IBtkα 1–1352) and pCMV6-XL5-Pdcd4 were from OriGene Technologies, Inc. (Rockville, MD). pcDNA3-Myc-Cul3 (plasmid 19893), pcDNA3-Myc-Cul3ΔN41 (plasmid 21590), pcDNA3-DN- hCul3-FLAG (plasmid 15820), and pcDNA3-HA2-Rbx1 (ROC1) (plasmid 19897) were from AddGene (Cambridge, MA). The pCMV-LUC and pCMV-SL-LUC plasmids were a kind gift from Dr. Hsin-Sheng Yang (Graduate Center for Toxicology, University of Kentucky, Lexington, KY). The prokaryotic expression vector of Pdcd4 wild type and mutants fused to GST (GST-Pdcd4-WT, GST-Pdcd4DRBD, or GST-Pdcd4RBDStop) were a kind gift of Dr. K. H. Klempnauer (Westfalische-Wilhelms-Universitat Munster).

GenScript Corp. (Piscataway, NJ) generated the following eukaryotic expression vectors of IBtkα mutants: pCMV6-IBtkαΔC-FLAG (aa 1–890), pCMV6-IBtkαΔN-FLAG (aa 307–1352), pCMV6-IBtkαΔBTB-FLAG (deletion of aa 564–836), pcDNA3.1(+)-Pdcd4-WT-HA, and pcDNA3.1(+)-Pdcd4 S67A/S71A/S76A.

ON-TARGET plus IBtkα siRNA, Cul3 siRNA, and control NO-TARGET siRNA were from GE Healthcare (Buckinghamshire, UK). ON-TARGET plus IBtkα siRNA includes a pool of siRNAs targeting the following sequences of IBtkα mRNA (NCBI reference sequence: XM_006715453.1): 2365–2474 (probe A002S42), 2400–2638 (probe D6S1188E), 4113–4214 (probe D6S1109E), and 5776–5879 (probe D6S1882).

The lentiviral constructs expressing the shRNA against IBtkα or control non-targeting shRNA (TRCN0000082575 and SHC002, respectively) were from MISSION® (Sigma-Aldrich). The shRNA-IBtkα targets the 2077–2098 nucleotides of IBtkα mRNA (NCBI reference sequence: XM_006715453.1). Lentiviral particles were produced in HEK293T cells, as described previously (28, 29).

Mouse anti-Pdcd4, mouse anti-HA, mouse anti-GAPDH, and mouse IgG antibodies were from Santa Cruz Biotechnology, Inc. Rabbit anti-Pdcd4, anti-Myc, anti-Ub Lys^48^, and anti-Ub Lys^63^ were from Cell Signaling Technology. Anti-Cul3 antibody was from BD Biosciences. Anti-FLAG was from Sigma-Aldrich. Anti-IBtk antibody was from Bethyl Laboratories, Inc. (Montgomery, TX).

##### Cell Lines, Transfection, and Treatments

HeLa and HEK293T cells were cultured in Dulbecco's modified Eagle's medium (Life Technologies, Inc.), supplemented with 10% heat-inactivated fetal calf serum, 2 mm l-glutamine and antibiotics (Life Technologies).

Cells were transfected with DNA using Lipofectamine 2000 (Life Technologies), according to the manufacturer's protocol. For siRNA, cells (3 × 10^6^) were transfected with 100 nmol of the indicated siRNA. When required, cells were treated with the proteasome inhibitor MG132 (Sigma-Aldrich), or protein biosynthesis inhibitor cycloheximide (CHX) (Sigma-Aldrich).

##### Cell Extracts, Immunoprecipitation (IP), and Western Blotting (WB)

Cells were lysed in modified RIPA buffer (10 mm Tris-HCl, pH 7.5, 150 mm NaCl, 1 mm EDTA, 1% Igepal). For IP, cells were lysed in RIPA buffer (50 mm Tris-HCl, pH 8.0, 150 mm NaCl, 1 mm EDTA, 1% Igepal, 0.5% sodium deoxycholate). Protein extraction was performed in the presence of protease inhibitor mixture (Roche Applied Science, Mannheim, Germany) and 2 mm *N*-ethylmaleimide (Sigma-Aldrich), using 1 ml of cold buffer for a 100-mm dish. Cell lysates were clarified by centrifugation at 14,000 × *g* for 10 min and then incubated overnight with the appropriate antibody, followed by a 2-h incubation with G-protein beads (30 μl/sample) (GE Healthcare). The beads were washed five times with 1 ml of cold RIPA buffer and denatured for 10 min at 70 °C in 25 μl of 2× NuPAGE sample buffer (Life Technologies). Protein samples were subjected to electrophoresis on NuPAGE 4–12% polyacrylamide gel (Life Technologies) or self-casted 6% polyacrylamide gel and then transferred onto a nitrocellulose membrane (GE Healthcare).

##### Mass Spectrometry

The pCMV6-IBtkα-FLAG plasmid and the corresponding empty vector were singularly transfected in HEK293T cells (24 μg of DNA/100-mm dish). Protein extracts (1.5 mg) from cells transfected with IBtkα-FLAG and empty vector were immunoprecipitated with anti-FLAG antibody (20 μg). Immunocomplexes were resolved by NuPAGE 4–12% SDS-PAGE, and gels were stained with colloidal Coomassie, as reported previously (30, 31). Protein bands were excised and subjected to in-gel tryptic digestion for mass spectrometry, according to Shevchenko *et al.* (32) and Käll *et al.* (33). Chromatography of tryptic peptides was performed on an Easy LC 1000 nanoscale liquid chromatography system (Thermo Fisher Scientific). The analytical nanoscale liquid chromatography column was a pulled fused silica capillary, 75-μm inner diameter, in-house packed to a length of 10 cm with 3-μm C18 silica particles from Dr. Maisch (Entringen, Germany). Peptide mixtures were loaded directly onto the analytical column at 500 nl/min. A binary gradient was used for peptide elution. Mobile phase A was 0.1% formic acid, 2% acetonitrile, whereas mobile phase B was 0.1% formic acid, 80% acetonitrile. Gradient elution was achieved at a 250 nl/min flow rate and ramped from 2% B to 45% B in 30 min. After 5 min at 100% B, the column was re-equilibrated at 2% B for 15 min before the following injection. MS detection was performed on a quadrupole-orbitrap mass spectrometer Q-Exactive (Thermo Fisher Scientific) operating in positive ion mode, with nanoelectrospray ionization potential at 1800 V applied on the column front-end via a tee-piece. Data-dependent acquisition was performed using a top-10 method. For full scans, resolution (full width at half-maximum) was set to 70,000, AGC target to 1e6, and maximum injection time to 50 ms, with an *m*/*z* range of 350–1800. MS/MS scans were acquired at 35,000 resolutions, 1e5 AGC target, and 150 ms maximum injection time. Mass window for precursor ion isolation was 1.6 *m*/*z*, whereas normalized collision energy was 25. Ion threshold for triggering MS/MS events was 2e4. Dynamic exclusion was 60 s. Data were processed by Proteome Discoverer version 1.3 (Thermo Fisher Scientific), using Sequest as a search engine, and the Swiss-Prot human database. The following search parameters were used: MS tolerance, 10 ppm; MS/MS tolerance, 0.02 Da; fixed modifications, carbamidomethyl cysteine; enzyme, trypsin; maximum missed cleavages, 2; taxonomy, human. High confidence peptides (confidence > 99%) were filtered out by using Percolator (48), integrated in Proteome Discoverer. Protein hits based on two successful peptide identifications were considered valid.

##### His and GST Pull-down

When indicated, ubiquitylated proteins were purified by histidine (His) pull-down. To this end, cells expressing His-tagged ubiquitin were lysed in denaturing buffer, containing 8 m urea, 100 mm Na_2_PO_4_, 10 mm Tris-HCl, pH 8.0. Lysates were applied to cobalt resin (Thermo Fisher Scientific) for 2 h, and then beads were washed twice with buffer containing 8 m urea, 100 mm Na_2_PO_4_, 10 mm Tris-HCl, pH 6.3; denatured in NuPAGE sample buffer; and loaded on a 6% polyacrylamide gel.

GST and GST-Pdcd4 recombinant proteins were produced in *Escherichia coli* BL21, as described previously (34). For GST pull-down, HEK293T cells (3 × 10^6^) were transfected with pCMV6-IBtkα-FLAG or IBtkα-FLAG mutant plasmids (4 μg), and 48 h later, cells were lysed in RIPA buffer. The lysate (1 mg) was incubated for 1 h with GST or GST-Pdcd4-WT and mutants at 4 °C under constant agitation. Then glutathione-Sepharose beads (30 μl) (GE Healthcare) were added to the mix for a 1-h incubation at 4 °C. Subsequently, beads were washed three times with RIPA buffer, and the bound proteins were eluted from beads by boiling in SDS sample buffer. Protein samples were subjected to electrophoresis on NuPAGE 4–12% polyacrylamide gel and analyzed by immunoblotting with the appropriate antibodies.

##### Protein Half-life

To measure the protein half-life, cells were treated with CHX (100 μg/ml) in culture medium, and WB of cell lysates (30 μg) was performed using the antibodies against Pdcd4, IBtkα, and GAPDH. Optical density of Pdcd4, IBtkα, and GAPDH WB protein bands was measured using ImageJ software; values of Pdcd4 or IBtkα bands were normalized to the GAPDH values. The normalized value of IBtkα and Pdcd4 at time 0 of the CHX time course experiments was taken as 100%), and the protein contents of the following time points were expressed as ratio of the indicated time point/time 0 (relative protein level). The ratio values were fitted by linear interpolation through the EXCEL program, and the corresponding equation was utilized to calculate the protein half-life. Densitometry was performed in at least three independent experiments.

##### Luciferase Assays

HeLa cells were transfected with siRNAs or expression vectors and cultured in DMEM medium supplemented with 10% FBS for 24 h. Then cells were retransfected with the report constructs pCMV-LUC and pCMV-SL-LUC. Cell lysates were analyzed for luciferase activity using the Dual-Luciferase assay kit (Promega), according to the manufacturer's instructions.

##### Protein Synthesis

HeLa cells (6 × 10^4^) were seeded into 24-well plates and 24 h later were incubated for 1 h in the presence of 50 μCi of ^35^S-labeled EasyTag Express protein labeling mix (PerkinElmer Life Sciences) in methionine-free medium supplemented with 10% dialyzed FCS. Then cells were lysed in RIPA buffer, and radiolabeled proteins were precipitated with trichloroacetic acid (TCA) on Whatman 3MM paper. The amount of radioactivity was determined by scintillation counting, and the counts were normalized to protein concentration.

##### Quantitative RT-PCR Analysis

Total RNA was extracted from cells with TRIzol reagent (Invitrogen). RNA aliquots (200 ng) were reverse transcribed with Random Examers (Roche Applied Science) and Superscript III Reverse Transcriptase (Invitrogen), according to the manufacturer's protocol. Real-time PCR was performed using iQ Green Super Mix (Bio-Rad) and carried out with the iCycler iQReal-Time detection system (Bio-Rad) under the following conditions: 95 °C for 1 min and then 40 cycles at 94 °C for 10 s and 60 °C for 30 s. Primers were as follows: IBtkα FW, 5′- GTCAGCCCTCCTGTTGTGGAT-3′; IBtkα REV, 5′-TGCATTCACTGGTTTGGGGGC-3′; Pdcd4 FW, 5′-CCATGGTGCTTCAATAGCATGT-3′; Pdcd4 REV, 5′-CCCAGCATTTTCTTCATCACCG-3′; Bcl-X_L_ FW, 5′-GTGAGTCGGATCGCAGCTT-3′; Bcl-X_L_ REV, 5′-GCTGCTGCATTGTTCCCATAG-3′; GAPDH FW, 5′-CAGCCTCAAGATCATCAGCA-3′; GAPDH REV, 5′-TGTGGTCATCAGTCCTTCCA-3′. Relative mRNA levels were normalized to the GAPDH level.

##### Statistical Analysis

Statistical analysis was performed by a paired two-tailed Student's *t* test. Differences were considered as statistically significant at the 95% level (*p* < 0.05).

##### Bioinformatics

Identity and similarity of human IBtkα and yeast Btb1 proteins were determined using the Clustal Omega program. Bioinformatics analysis for structural identification and representation of the 3-box motif of IBtkα (amino acids 861–901) was carried out using the Phyre2 computational program (35).

## Results

### 

#### 

##### IBtkα Is a Component of Cul3-dependent E3 Ligase

**FIGURE 1.**
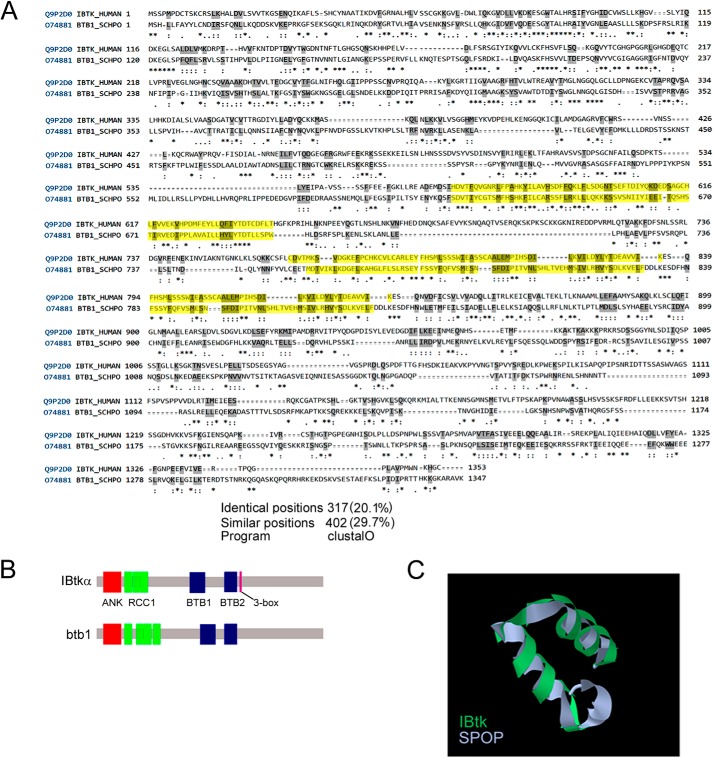
*A*, Clustal Omega program-based analysis of identity and similarity of human IBtkα and yeast Btb1 proteins. *Yellow boxes* highlight the BTB domains. *, identical positions, which have a single, fully conserved residue; *colon* and *period*, similar positions, which have conservation between groups of amino acids with strongly or weakly similar properties, respectively. *B*, schematic representation of the domain organization of human IBtkα and yeast Btb1 proteins (data from SwissProt, InterPro). *C*, superposition of the 3-box motifs of IBtkα and SPOP was elaborated by using Phyre2 software.

Clustal Omega multiple sequence alignment program-based bioinformatics analysis identified a significant homology of human IBtkα (UniProtKB Q9P2D0) and *S. pombe* Btb1 (UniProtKB O74881) with similarity and identity in amino acid positions of 30 and 20%, respectively (Fig. 1*A*). Domain organization is strictly conserved in the two proteins because they contain two ankyrin repeats at the N terminus followed by a tandem array of RCC1 domains and two separated BTB domains (Fig. 1*B*). Structural homology and fold recognition analysis carried out by the Phyre2 computational tool identified a 3-box motif placed at the C terminus of BTB2 domain, which significantly matched with the 3-box motif of different BTB proteins, such as SPOP, gigaxonin, KLH4, KLH11, and BTBD6 (Table 1); the highest structural homology was with the 3-box motif of SPOP (confidence 98.2%) (Table 1). Because *S. pombe* Btb1 interacts with Pcu3p, the yeast orthologue of human Cul3, and is substrate receptor of Pcu3p(Cul3)-based ubiquitin ligase (11, 14), we speculated that IBtkα could be a substrate receptor of human CRL3.

**TABLE 1:** **BTB proteins containing a 3-box motif with high structural homology with the 3-box motif of IBtkα, as resulted from the Phyre2 computational tool-based bioinformatics analysis of secondary structure** The % identity column indicates the percentage identity between the C-terminal region of IBtkα BTB2 domain (aa 861–901) and the matched BTB protein. Confidence indicates the probability (from 0 to 100) that the match between the IBtkα sequence and the BTB protein is a true homology.

BTB proteins	% identity	Confidence	UniProt accession
	%		
Specle-type POZ protein	41	98.2	O43791
Gigaxonin	15	96.8	Q9H2C0
Kelch repeat and BTB domain-containing protein 4	11	96.4	Q9NVX7
Kelch-like protein 11	32	96.4	Q9NVR0
BTB/POZ domain-containing protein 6	21	90.8	Q96KE9

**FIGURE 2.**
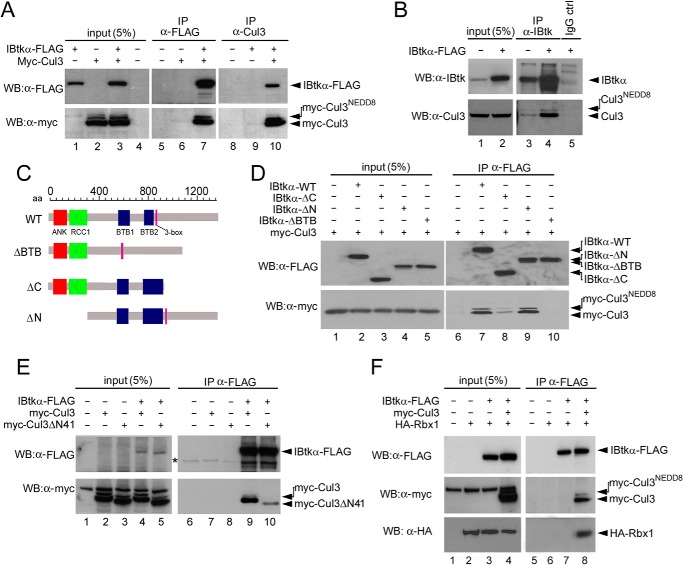
**IBtkα assembles within a CRL3 ubiquitin ligase complex *in vivo*.** *A*, association of ectopically expressed IBtkα and Cul3. HEK293T cells (3 × 10^6^) were transfected with IBtkα-FLAG (4 μg) or Myc-Cul3 (4 μg) or left untransfected. Forty-eight hours later, cell extracts (1 mg) were immunoprecipitated with the indicated antibodies and analyzed by WB. *B*, association of endogenous IBtkα and Cul3. HeLa cells (3 × 10^6^) were transfected with IBtkα-FLAG (4 μg) or left untransfected. The following steps were performed as indicated in *A. C*, schematic representation of IBtkα mutants used in this study. *D*, BTB domains of IBtkα are required for binding to Cul3. HEK293T cells (3 × 10^6^) were transfected with Myc-Cul3 (4 μg) and wild type or mutant IBtkα-FLAG (4 μg) or left untransfected. The following steps were performed as indicated in *A. E*, IBtkα binds to the amino terminus of Cul3. HEK293T cells (3 × 10^6^) were transfected with IBtkα-FLAG (4 μg), Myc-Cul3 (4 μg), or Myc-Cul3ΔN41 (4 μg) or left untransfected. The following steps were performed as indicated in *A*. *, nonspecific bands. *F*, IBtkα generates a macromolecular complex with Cul3 and Rbx1. HEK293T (3 × 10^6^) cells were transfected with IBtkα-FLAG (4 μg), Myc-Cul3 (4 μg), and HA-Rbx1 (4 μg). The following steps were performed as indicated in *A*.

We first investigated the *in viv*o interaction of IBtkα with Cul3. HEK293T cells were transfected with Myc-Cul3 in the presence or absence of IBtkα-FLAG, and 48 h later, cell extracts were immunoprecipitated with anti-FLAG or anti-Cul3 antibodies, followed by WB analysis of immunocomplexes. Myc-Cul3 was detected in the IBtkα-FLAG immunocomplex as two tandem protein bands with the expected molecular mass of about 90 kDa (Fig. 2*A*, *lane 7*), the slower band being the Cul3 neddylated form; as a control, Myc-Cul3 was not immunoprecipitated in Myc-Cul3-transfected cells in the absence of IBtkα-FLAG (Fig. 2*A*, *lane 6*). Similarly, IBtkα-FLAG was specifically detected in Myc-Cul3 immunocomplex (Fig. 2*A*, *lane 10*). As an additional experiment, the association of endogenous Cul3 with transfected IBtkα-FLAG or endogenous IBtkα was observed by IP of cell extracts with anti-IBtkα antibody and not control IgG (Fig. 2*B*, *lanes 3* and *4*). Altogether, these results indicated that IBtkα *in vivo* associated with Cul3.

Next, we mapped the amino acid sequence of IBtkα and Cul3 required for the association of the two proteins. The mutant IBtkαΔC, lacking the C-terminal region including the 3-box motif (deletion of aa 891–1352; Fig. 2*C*), weakly bound to Myc-Cul3 (Fig. 2*D*, compare *lanes 7* and *8*). The mutant IBtkαΔN, lacking the ankyrin repeats and RCC1 domains (deletion of aa 1–307; Fig. 2*C*), was able to bind to Myc-Cul3 as efficiently as the wild type (Fig. 2*D*, compare *lanes 7* and *9*). Conversely, the mutant IBtkαΔBTB, which lacked the BTB1 and BTB2 domains (deletion of aa 564–836; Fig. 2*C*) did not associate with Myc-Cul3 (Fig. 2*D*, compare *lanes 7* and *10*), which was which is consistent with the requirement of a BTB domain for binding to Cul3 (11). The N-terminal region of Cul3 was previously reported to interact with the BTB domain (20). Consistently, the mutant Cul3ΔN41 (deletion of 41 amino acids at the N terminus) (20) weakly coimmunoprecipitated with IBtkα-FLAG (Fig. 2*E*, compare *lanes 9* and *10*). Altogether, these results indicated that the IBtkα region, including the two BTB domains and 3-box motif, was required for the binding to the N terminus of Cul3.

To verify whether IBtkα was a component of a canonical CRL3, we tested whether the *in vivo* generated IBtkα-Cul3 complex included Rbx1, which is the CRL3 component recruiting the Ub-charged E2 enzyme (4). To this end, HEK293T cells were transfected with HA-Rbx1 in the presence or absence of IBtkα-FLAG and Myc-Cul3 expression vectors, and IBtkα-FLAG was immunoprecipitated with anti-FLAG antibody. HA-Rbx1 was detected in the IBtkα-FLAG immunocomplex in the presence of ectopically expressed Myc-Cul3 (Fig. 2*F*, *lane 8*), whereas it was undetected in the absence of IBtkα-FLAG and Myc-Cul3 (Fig. 2*F*, *lanes 6* and *7*). Overall, these results indicated that IBtkα was a component of a CRL3 complex including Cul3 and Rbx1.

##### IBtkα Is Autoubiquitylated through CRL3

**FIGURE 3.**
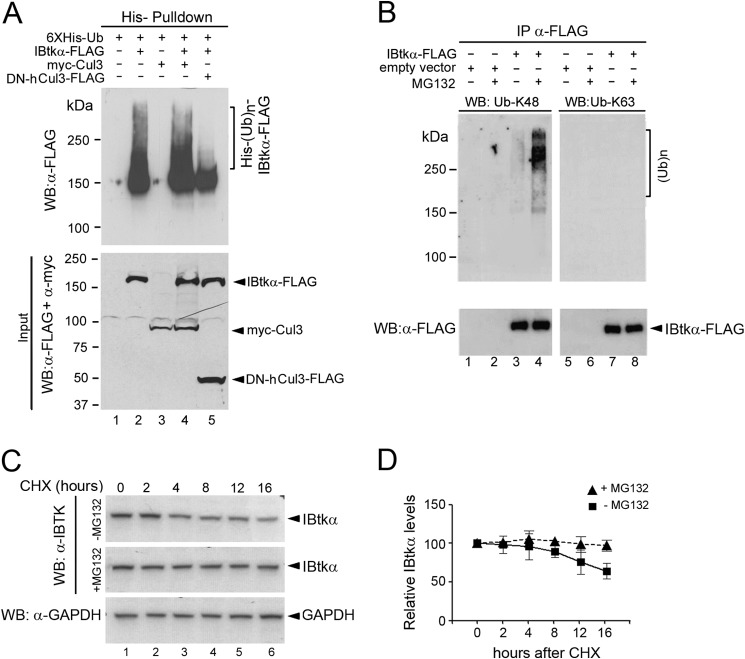
**IBtkα undergoes Cul3-dependent Lys^48^ polyubiquitylation and proteasomal degradation.** *A*, Cul3 mediates the polyubiquitylation of IBtkα. HEK293T cells (3 × 10^6^) were transfected with His-tagged ubiquitin (His-Ub; 12 μg), with or without IBtkα-FLAG (4 μg), Myc-Cul3 (4 μg), or DN-hCul3-FLAG (4 μg) expression vectors. Forty-eight hours after transfection, cells were treated with MG132 (20 μm) for 4 h before lysis. Cell extracts were pulled down with cobalt-agarose affinity chromatography, resolved by 6% SDS-PAGE, and analyzed by WB with anti-FLAG antibody to detect IBtkα-FLAG ubiquitylated isoforms. *B*, IBtkα undergoes Lys^48^ polyubiquitylation *in vivo*. HEK293T cells (3 × 10^6^) were transfected with IBtkα-FLAG (4 μg) or the corresponding empty vector (4 μg), and 48 h later, cells were treated with MG132 (20 μm) for 4 h before lysis. Cell extracts were immunoprecipitated with anti-FLAG antibody and resolved by 6% SDS-PAGE, followed by immunoblotting with anti-Ub Lys^48^ or anti-Ub Lys^63^ antibody. *C*, evaluation of IBtkα half-life. HEK293T cells (3 × 10^6^) were treated for 1 h with MG132 (20 μm) or vehicle and then incubated with CHX (100 μg/ml) for up to 16 h. Cell lysates (30 μg) were separated by NuPAGE 4–12% SDS-PAGE and analyzed by WB with the indicated antibodies. *D*, quantification of IBtkα half-life. Protein band intensities of the experiment described in *C* were normalized to the corresponding GAPDH intensity and then normalized to the 0 h time point (100%). The mean densitometric values ± S.D. (*error bars*) of three independent experiments are shown.

A characteristic signature of most E3s is the ability to catalyze autoubiquitylation, which is usually assayed to confirm that proteins assemble into an active ubiquitin ligase complex (36). To assess whether IBtkα was ubiquitylated *in vivo*, HEK293T cells were transfected with the expression vector of IBtkα-FLAG and His-tagged ubiquitin, and the ubiquitylated proteins were pulled down and analyzed by WB with anti-FLAG antibody. Polyubiquitylated IBtkα-FLAG isoforms were observed (Fig. 3*A*, *lane 2*), which were slightly increased by overexpression of wild type Myc-Cul3 (Fig. 3*A*, *lane 4*) and almost abolished by overexpression of the dominant negative mutant DN-hCul3-FLAG (Fig. 3*A*, *lane 5*), which is devoid of the binding site for Rbx1, and thus inhibits the CRL3 catalytic activity (19). To analyze the pattern of IBtkα polyubiquitylation, HEK293T cells were transfected with the expression vector of IBtkα-FLAG or empty vector, and cell lysates were immunoprecipitated with anti-FLAG antibody and analyzed by WB with anti-Ub Lys^48^ or anti-Ub Lys^63^ antibodies. We observed Lys^48^ polyubiquitylation of IBtkα-FLAG that was strongly enhanced by cell treatment with the 26S proteasome inhibitor MG132 (Fig. 3*B*). No significant Lys^63^ ubiquitination of IBtkα-FLAG was detected (Fig. 3*B*, *lanes 7* and *8*). The half-life of IBtkα was determined in the presence or absence of MG132 by blocking *ex novo* protein synthesis with CHX. The IBtkα protein level slightly decreased in the absence of MG132 with an estimated half-life of about 24 h, whereas it was unaffected in the presence of MG132 (Fig. 3, *C* and *D*). Overall, these results confirmed that IBtkα was a component of a Cul3-dependent ubiquitin ligase complex, thereafter named CRL3^IBTK^, and underwent Cul3-dependent Lys^48^ polyubiquitylation coupled to proteasomal degradation.

##### IBtkα Physically Interacts with Pdcd4

**FIGURE 4.**
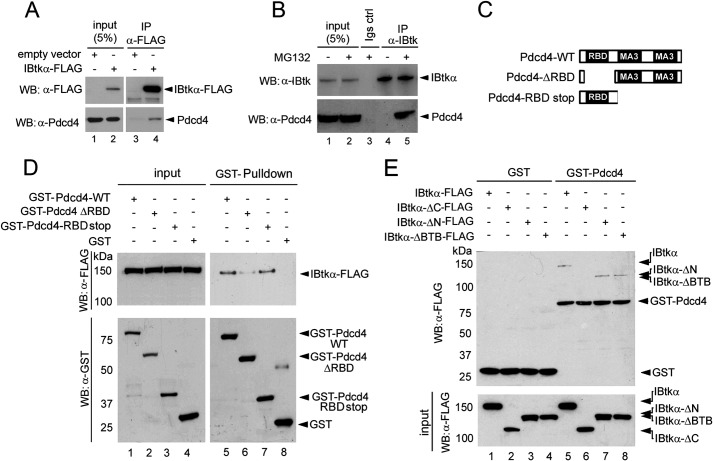
**IBtkα interacts with Pdcd4.** *A*, *in vivo* association of IBtkα-FLAG with Pdcd4. HEK293T cells (3 × 10^6^) were transfected with IBtkα-FLAG or empty vector (4 μg). Forty-eight hours after transfection, cell lysates were immunoprecipitated with anti-FLAG antibody, and immunocomplexes were analyzed by WB with the indicated antibodies. *B*, *in vivo* association of endogenous IBtkα and Pdcd4 proteins. HEK293T cells (3 × 10^6^) were treated for 4 h with MG132 (20 μm) or vehicle. Cell lysates were immunoprecipitated with anti-IBtk or IgG antibodies, and immunocomplexes were analyzed by WB with the indicated antibodies. *C*, schematic representation of Pdcd4 wild type and mutants. *D*, mapping of Pdcd4 binding site for IBtkα. HEK293T cells (3 × 10^6^) were transfected with IBtkα-FLAG (4 μg), and cell lysates were subjected to GST pull-down, using GST-Pdcd4-WT, GST-Pdcd4DRBD, or GST-Pdcd4RBDStop recombinant proteins as baits. The pull-down was analyzed by WB with the indicated antibodies. *E*, mapping of IBtkα binding site for Pdcd4. HEK293T cells (3 × 10^6^) were transfected with IBtkα-FLAG or IBtkα-FLAG deletion mutants (4 μg). Cell lysates were subjected to GST-Pdcd4 pull-down and analyzed by WB with anti-FLAG antibody.

To identify putative substrates of CRL3^IBTK^, we used a strategy based on immunopurification of ectopically expressed IBtkα-FLAG in HEK293T cells, followed by mass spectrometry of IBtkα-FLAG immunocomplex. We identified several IBtkα interactors that are involved in the ubiquitin-proteasome system pathway, including Cul3; UBA1 (ubiquitin-activating enzyme E1); ubiquitin; and the classical CRL regulatory proteins NEDD8, CAND1, and subunit protein of the COP9 signalosome complex (Table 2). These findings supported a role of CRL3^IBTK^ in the ubiquitin-proteasome system. We also identified the Pdcd4 (programmed cell death 4) protein, a well known tumor suppressor involved in several cellular processes (37). The association of endogenous Pdcd4 with transfected IBtkα-FLAG was proved by co-IP of cell extracts with anti-FLAG antibody (Fig. 4*A*). Co-IP of endogenous Pdcd4 and IBtkα proteins was observed using the anti-IBtkα antibody in the presence of the proteasome inhibitor MG132 (Fig. 4*B*).

**TABLE 2:** **Mass spectrometry-based identification of proteins coimmunoprecipitated *in vivo* with IBtkα-FLAG** Coverage indicates the percentage of the amino acid sequence that overlaps with the identified peptide species.

Accession no.	Description	Coverage	Unique peptides
Q9P2D0	Inhibitor of Bruton tyrosine kinase	60.16	110
Q86VP6	Cullin-associated NEDD8-dissociated protein 1	10.81	12
P61201	COP9 signalosome complex subunit 2	22.35	11
B3KST5	COP9 signalosome complex subunit 4	30.40	11
P22314	Ubiquitin-like modifier-activating enzyme 1	8.22	11
Q13618	Cullin3	6.99	6
F5H265	Polyubiquitin-C	63.09	22
Q9UNS2-2	COP9 signalosome complex subunit 3	11.66	5
Q53EL6	Programmed cell death protein 4	12.90	3

Pdcd4 contains an N-terminal RNA-binding domain and two MA-3 domains located in the central and C-terminal regions (Fig. 4*C*) (38). To map the amino acid sequence of Pdcd4 required for the binding to IBtkα, wild type and Pdcd4 mutants were used in GST pull-down experiments of cell extracts from IBtkα-FLAG-transfected HEK293T (Fig. 4*C*). Consistently with *in vivo* co-IP, IBtkα was pulled down by GST-Pdcd4-WT (Fig. 4*D*, *lane 5*). Conversely, IBtkα was slightly pulled down by the Pdcd4-ΔRBD mutant, which lacks the RNA-binding domain (Fig. 4*D*, *lane 6*), indicating that the RNA-binding domain of Pdcd4 was required for the binding to IBtkα. Differently, IBtkα was fully recovered by GST-Pdcd4-RBDStop pull-down (Fig. 4*D*, *lane 7*), indicating that the MA3 domains of Pdcd4, lacking in the Pdcd4-RBDStop mutant, were dispensable for the binding to IBtkα. By GST pull-down, we also mapped the IBtkα domains required for the binding to Pdcd4, using HEK293 transfected with IBtkα-FLAG mutants. IBtkαΔN-FLAG and IBtkαΔBTB-FLAG were recovered by GST-Pdcd4-WT pull-down (Fig. 4*E*, *lanes 7* and *8*), indicating that the region encompassing the ankyrin, RCC1, and BTB domains was dispensable for the binding to Pdcd4. Conversely, IBtkαΔC-FLAG did not bind to GST-Pdcd4-WT (Fig. 4*E*, *lane 6*), indicating that the C-terminal region of IBtkα, encompassing amino acids 891–1353, included the IBtkα binding site to Pdcd4.

##### IBtkα Promotes the Ubiquitylation Coupled to Proteasomal Degradation of Pdcd4

**FIGURE 5.**
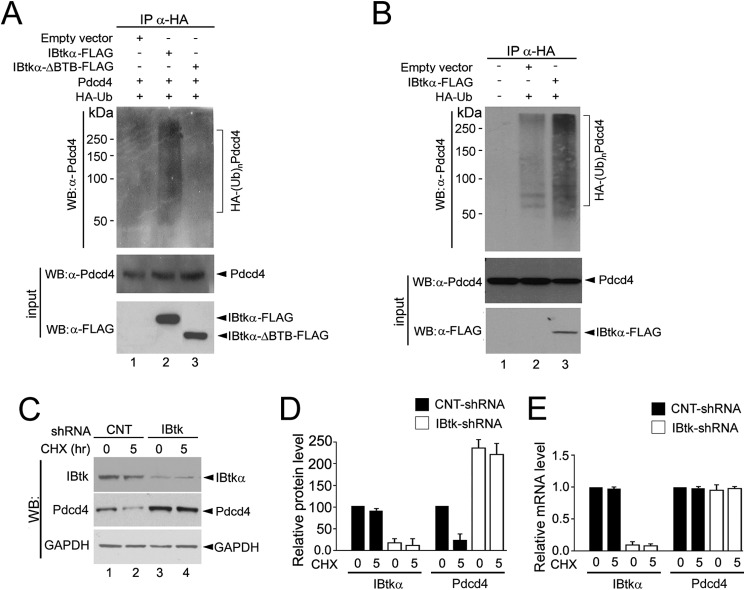
**IBtkα promotes the Pdcd4 polyubiquitylation and degradation.** *A*, IBtkα promotes the polyubiquitylation of exogenously expressed Pdcd4. HEK293T cells (3 × 10^6^) were transfected with Pdcd4 (4 μg) with or without IBtkα-FLAG, IBtkαΔBTB-FLAG, HA-tagged ubiquitin, or empty vector (4 μg). Forty-eight hours later, cells were treated with MG132 (20 μm) for 4 h before lysis. Cell extracts were subjected to IP with anti-HA antibody, and immunocomplexes were resolved by SDS-PAGE on a 6% gel, followed by WB with the indicated antibodies. *B*, IBtkα promotes the polyubiquitylation of endogenous Pdcd4 *in vivo. C*, IBtkα RNA interference increases the Pdcd4 protein content. HeLa cells (3 × 10^6^) were transduced with control shRNA (*CNT-shRNA*), or IBtk shRNA for 48 h and then incubated with CHX (100 μg/ml) for up to 5 h. Cell lysates (30 μg) were analyzed by WB with the indicated antibodies. *D*, densitometric analysis of WB protein bands relative to the experiment described in *C*. Optical density of WB protein bands was expressed as arbitrary units normalized to control shRNA taken as a value of 100. Mean values ± S.D. (*error bars*) of three independent experiments are shown. *E*, IBtkα RNA interference does not affect the Pdcd4 mRNA level. HeLa cells were transduced as described in *C*, total RNA was extracted 48 h later, and IBtkα and Pdcd4 transcripts were measured by quantitative RT-PCR. Relative mRNA levels were expressed as arbitrary units normalized to control shRNA taken as 1.0. Mean values ± S.D. of three independent experiments are shown.

Next, we tested the hypothesis that IBtkα, as CRL3 component and Pdcd4 interactor, could promote Pdcd4 ubiquitylation coupled to proteasomal degradation. By a ubiquitylation assay *in vivo*, we observed polyubiquitylated Pdcd4 isoforms in empty vector-transfected HEK293T cells, whose detection was increased by IBtkα-FLAG transfection (Fig. 5*A*, compare *lanes 1* and *2*) and reduced by the mutant IBtkαΔBTB-FLAG, which binds to Pdcd4 and not to Cul3 (Fig. 5*A*, *lane 3*), supporting the requirement of IBtkα interaction with Cul3 to promote Pdcd4 polyubiquitylation. Further, the overexpression of IBtkα-FLAG also increased the polyubiquitylation of endogenous Pdcd4 (Fig. 5*B*). Moreover, depletion of IBtkα by RNA interference increased the steady-state Pdcd4 protein level (Fig. 5, *C* (compare *lanes 1* and *3*) and *D*) without significantly affecting the *PDCD4* mRNA level (Fig. 5*E*). The inhibition of protein synthesis with CHX caused a large reduction of Pdcd4 protein level in control shRNA-treated cells (Fig. 5*C*, *lanes 1* and *2*), which is consistent with the short half-life of Pdcd4 protein (39). Conversely, Pdcd4 protein level was unaffected in IBtkα depleted and CHX-treated cells (Fig. 5*C*, *lanes 3* and *4*), indicating that IBtkα affected the protein amount of Pdcd4 acting at a post-transcriptional level.

**FIGURE 6.**
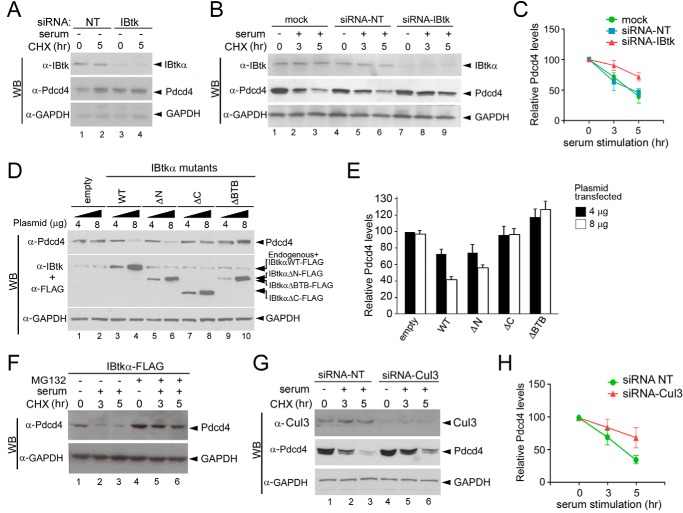
**IBtkα promotes the Pdcd4 proteasomal degradation upon serum stimulation.** *A*, serum deprivation does not affect the IBtkα-dependent Pdcd4 degradation. HeLa cells (3 × 10^6^) were transfected with IBtk siRNA or control siRNA (*siRNA-NT*). Forty-eight hours later, cells were incubated with CHX (100 μg/ml) and serum-starved for up to 5 h. Cell lysates (30 μg) were separated by 4–12% NuPAGE and analyzed by WB with the indicated antibodies. *B*, IBtkα RNA interference counteracts the serum-induced Pdcd4 degradation in starved cells. HeLa cells (3 × 10^6^) were transfected with IBtk siRNA, control siRNA, or left untransfected (mock). Forty-eight hours later, cells were serum-starved for 16 h (time point 0) and then replenished with serum (10%) and incubated with CHX (100 μg/ml) for up to 5 h. The following steps were as described in *A. C*, quantification of Pdcd4 half-life with or without IBtkα RNA interference upon serum addition in starved cells. Protein band intensities of the experiment described in *B* were normalized to the corresponding GAPDH intensity and then compared with the 0 h time point. The mean densitometric values ± S.D. (*error bars*) of three independent experiments are shown. *D*, overexpression of IBtkα augments the degradation of Pdcd4. HeLa cells (3 × 10^6^) were transfected with two amounts (4 or 8 μg) of IBtkα-FLAG or deletion mutants or empty vector. Forty-eight hours later, cell lysates (30 μg) were separated by NuPAGE 4–12% and analyzed by WB with the indicated antibodies. *E*, quantification of the Pdcd4 level of the experiment described in *D*. Protein bands were normalized to the corresponding GAPDH intensity. The mean densitometric values ± S.D. of three independent experiments are shown. *F*, MG132 proteasome inhibitor rescues Pdcd4 from IBtkα-mediated degradation. HeLa cells (3 × 10^6^) were transfected with IBtkα-FLAG or empty vector (4 μg) and 48 h later were serum-starved for 16 h and then replenished with serum (10%) and incubated with MG132 (20 μm) for up to 5 h. *G*, Cul3 RNA interference counteracts the serum-induced Pdcd4 degradation. HeLa cells (3 × 10^6^) were transfected with Cul3 siRNA or control siRNA and subjected to serum starvation/addition and CHX treatment, as detailed in *B*. Cell extracts were analyzed by WB with the indicated antibodies. *H*, quantification of Pdcd4 half-life of the experiment described in *G*. Protein bands were normalized to the corresponding GAPDH intensity and then compared with the 0 h time point. The mean densitometric values ± S.D. of three independent experiments are shown.

Pdcd4 is strictly regulated by serum signaling (39, 40) and undergoes CRL1-mediated ubiquitylation and proteasomal degradation upon serum replenishment in serum-starved cells (39). Thus, we tested whether IBtkα could affect the stability of Pdcd4 in response to serum. To this end, the Pdcd4 protein content was analyzed in CHX time course experiments, where HeLa cells were serum-starved for 16 h and then replenished with serum for the following 5 h, with or without IBtkα RNA interference. Consistent with a previous report (39), Pdcd4 accumulated upon serum starvation as compared with normal serum condition in control siRNA-transfected cells (Fig. 6*A*, *lanes 1* and *2*). Conversely, Pdcd4 accumulation was equally observed in IBtkα siRNA-transfected cells with or without serum starvation (Fig. 6*A*, *lanes 3* and *4*). When serum was added to starved cells, a slower rate of degradation of Pdcd4 was observed in IBtkα siRNA-transfected cells as compared with untransfected and control siRNA-transfected cells, with a Pdcd4 half-life of 9.2, 2.9, and 3.2 h, respectively (Fig. 6, *B* and *C*). These results clearly indicated that IBtkα mediated the Pdcd4 degradation in response to serum because the lack of IBtkα interfered with the serum-induced degradation of Pdcd4. Moreover, the transfection of wild type IBtkα and IBtkαΔN-FLAG increased the degradation of Pdcd4 as compared with empty vector (Fig. 5, *D* (*lanes 1–6*) and *E*), whereas the mutant IBtkαΔC did not (Figs. 5*E* and 6*D* (*lanes 7* and *8*)). Conversely, the transfection of IBtkαΔBTB-FLAG resulted in a slight increase of Pdcd4 (Figs. 5*E* and 6*D* (*lanes 9* and *10*)), indicating that IBtkαΔBTB behaved as a dominant negative mutant. These results indicated that the amino acid sequences of IBtkα containing the BTB domains for Cul3 binding and the C terminus for Pdcd4 binding were both required for serum-induced degradation of Pdcd4. Further, MG132 treatment prevented the degradation of Pdcd4 in IBtkα-FLAG-transfected cells (Fig. 6*F*), indicating that IBtkα promoted the proteasomal degradation of Pdcd4. Finally, Cul3 RNA interference increased the Pdcd4 half-life as compared with siRNA control, indicating the involvement of Cul3 in Pdcd4 degradation (Fig. 6, *G* and *H*).

**FIGURE 7.**
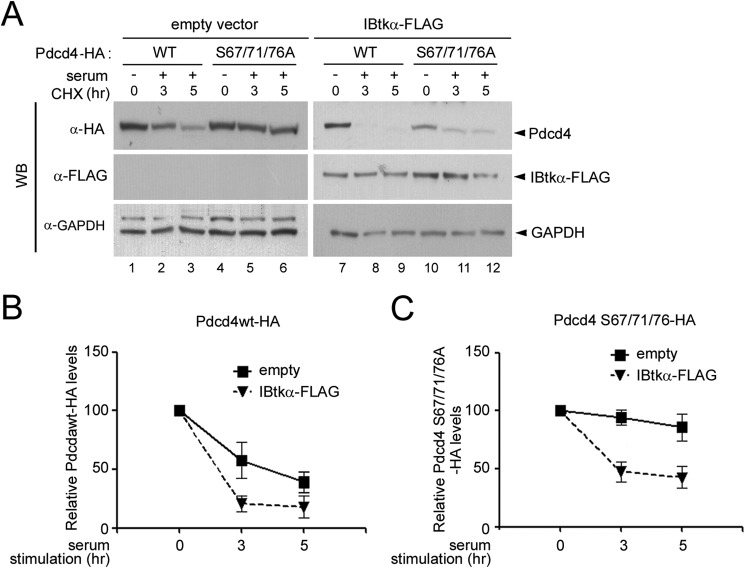
**IBtkα-mediated ubiquitylation of Pdcd4 does not depend on serines 71 and 76.** *A*, overexpression of IBtkα induces the degradation of Pdcd4 S67A/S71A/S76A-HA mutant. HeLa cells (3 × 10^6^) cells were transfected with Pdcd4-WT-HA (2 μg) or Pdcd4 S67A/S71A/S76A-HA (2 μg) expression vectors, with or without IBtkα-FLAG (4 μg) or empty (4 μg) vector. Forty-eight hours later, cells were serum-starved for 16 h (time point 0) and then replenished with serum (10%) and incubated with CHX (100 μg/ml) for up to 5 h. Cell lysates (30 μg) were separated by NuPAGE 4–12% and analyzed by WB with the indicated antibodies. *B* and *C*, quantification of Pdcd4-WT-HA (*B*) or Pdcd4 S67A/S71A/S76A-HA (*C*) half-life with or without IBtkα overexpression upon serum addition in starved cells. Protein band intensities of the experiment described in *A* were normalized to the corresponding GAPDH intensity and then compared with the 0 h time point. The mean densitometric values ± S.D. (*error bars*) of three independent experiments are shown.

The CRL1-mediated ubiquitylation of Pdcd4 depends on its phosphorylation at serine 67, 71, and 76 and is mediated by the βTrcp substrate receptor of the SCF^βTrcp^ E3 ligase (39). Thus, we addressed the role of serine 67, 71, and 76 on IBtkα-mediated ubiquitylation of Pdcd4 by analyzing the stability of the exogenously expressed Pdcd4-WT-HA or Pdcd4 S67A/S71A/S76A-HA mutant, which is resistant to the SCF^βTrcp^-mediated ubiquitylation/degradation (39), in CHX time course experiments. Upon serum deprivation/replenishment, the exogenously expressed Pdcd4-WT-HA protein was degraded at a rate similar to that observed for endogenous Pdcd4 (Fig. 7, *A* and *B*), whereas the Pdcd4 S67A/S71A/S76A-HA mutant was resistant to the serum-induced degradation (Fig. 7, *A* and *C*), consistent with a previous report (39). Surprisingly, the overexpression of IBtkα-FLAG significantly increased the degradation of both the Pdcd4-WT-HA and the Pdcd4 S67A/S71A/S76A-HA mutant upon deprivation/replenishment (Fig. 7, *A–C*). This result indicates that IBtkα-mediated ubiquitylation of Pdcd4 does not require the presence of serine 67, 71, and 76.

##### IBtkα Modulates the Translational Activity of Pdcd4

**FIGURE 8.**
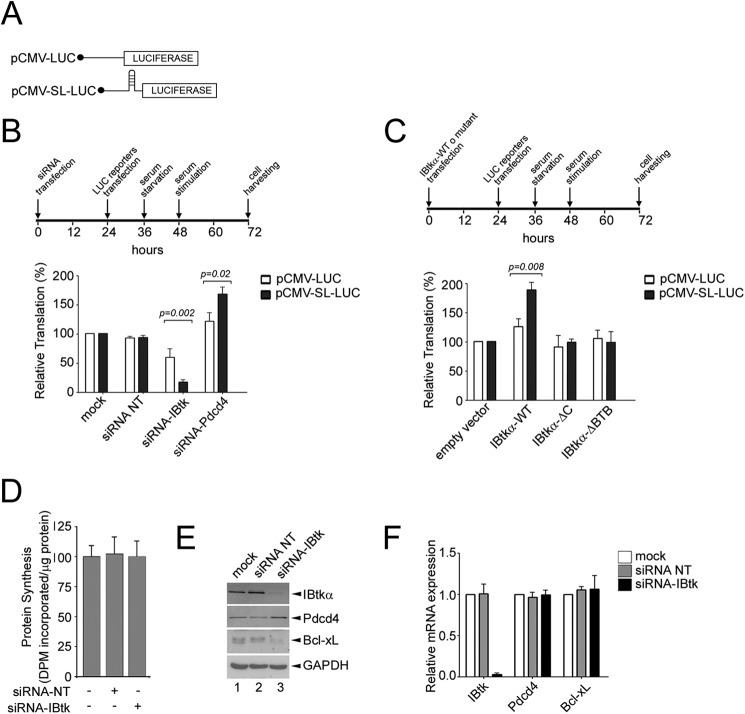
**IBtkα enhances translation by counteracting the Pdcd4 repression of target mRNAs.** *A*, schematic representation of luciferase reporter mRNAs. *B*, depletion of IBtkα by RNA interference decreases the translation of reporter mRNAs with stem-loop structured or unstructured 5′-UTR. HeLa cells (3 × 10^6^) cells were transfected with IBtk siRNA, Pdcd4 siRNA, or control siRNA or left untransfected (*mock*). After 24 h, cells were transfected with pCMV-LUC (0.2 μg) or pCMV-SL-LUC (0.2 μg) and serum-starved for 12 h, followed by growth in complete medium (10% FBS) for additional 24 h. The luciferase activity measured in untransfected cells was designated as 100%. Mean values ± S.D. (*error bars*) of five independent experiments are shown. *C*, overexpression of IBtkα enhances the translation of reporter mRNAs with stem-loop structured or unstructured 5′-UTR. HeLa cells (3 × 10^6^) were transfected with IBtkα-FLAG, IBtkα-FLAG mutants, or empty vector (4 μg). Subsequent steps were performed as described in *B. D*, IBtkα RNA interference does not affect the global protein synthesis. HeLa cells (3 × 10^6^) were transfected with IBtk siRNA or control siRNA or left untransfected (*mock*). The rate of protein synthesis was measured by incorporation of ^35^S-labeled methionine and cysteine into translated protein and normalized to total protein concentration. *E*, IBtkα depletion by RNA interference decreases the intracellular amount of Bcl-xL. HeLa cells (3 × 10^6^) were transfected with IBtk siRNA or control siRNA or left untransfected (*mock*), and 48 h later, cell lysates were analyzed by WB with the indicated antibodies. *F*, HeLa cells were transfected as described in *E*, and total RNA was analyzed by real-time PCR to measure the level of the indicated transcripts. Mean values ± S.D. of three independent experiments are shown.

Pdcd4 inhibits the translation of mRNAs with structured 5′-UTR by repressing the eIF4A1 helicase activity (40, 41). In addition, Pdcd4 inhibits the IRES-dependent translation of antiapoptotic proteins, such as Bcl-xL,XIAP (42) and c-Myb (38) through direct binding to the IRES region. Thus, we investigated whether IBtkα affected the Pdcd4-dependent translation by regulating the Pdcd4 stability. To this end, we performed an *in vivo* translation assay using two luciferase mRNA reporters containing either a stable stem-loop in the 5′-UTR or an unstructured 5′-UTR (Fig. 8*A*). By use of this experimental system, Pdcd4 was previously shown to preferentially inhibit the translation of mRNA with stem-loop structured 5′-UTR (41). As compared with mock and siRNA control, depletion of IBtkα by RNA interference reduced the translation of both mRNA reporters, with a more significant decrease of stem-loop structured 5′-UTR (Fig. 8*B*). Conversely, depletion of Pdcd4 significantly increased the translation of both mRNA luciferase reporters (Fig. 8*B*).

As an additional experiment, the transfection of IBtkα-FLAG also increased the translation of both mRNA luciferase reporters, with a greater increase of stem-loop structured 5′-UTR (Fig. 8*C*). Moreover, IBtkαΔC and IBtkαΔBTB mutants, lacking the IBtkα binding sites for Pdcd4 and Cul3, respectively, did not affect the translation of the luciferase reporters (Fig. 8*C*). In the context of the luciferase reporter system, these results indicated that IBtkα promoted translation with a preferential effect on mRNA endowed with stem loop structured 5′-UTR, being the IBtkα interaction domains with Pdcd4 and Cul3 required for this action.

We also determined whether IBtkα affected the global protein synthesis and the translation of Bcl-X_L_ as a Pdcd4-specific mRNA target (42). In HeLa cells, IBtkα depletion by RNA interference did not modify the rate of global protein synthesis (Fig. 8*D*); however, it significantly decreased the Bcl-X_L_ protein content while increasing Pdcd4 (Fig. 8*E*). As control, the levels of Bcl-X_L_ and Pdcd4 transcripts were unaffected by IBtkα RNA interference (Fig. 8*F*). These results indicated that IBtkα enhanced the translation of a Pdcd4-dependent transcript, such as Bcl-X_L_, by affecting the Pdcd4 stability.

## Discussion

BTB proteins can regulate several cellular processes by promoting the recruitment of degradation targets to E3 ubiquitin ligase complexes. In this study, we have demonstrated that IBtkα, an uncharacterized BTB protein, is substrate receptor of a Cul3-dependent ubiquitin ligase, here named CRL3^IBTK^. In fact, we have demonstrated the *in vivo* association of IBtkα with Cul3 by co-immunoprecipitation of endogenous or ectopically expressed proteins in HEK293T cells, which is consistent with previous reports on physical interaction of IBtkα with Cul3 in 293T cells (43), mouse embryonic stem cells (44, 45), and NKT lymphocytes (44). Then we have shown that the BTB domains of IBtkα and the N terminus of Cul3 mediated the association of the two proteins, which is consistent with the requirement of the BTB domain for binding to the N terminus of Cul3 (11, 13). In addition, we showed that the 3-box motif at the C-terminal side of the IBtkα BTB2 domain stabilized the binding of IBtkα to Cul3, which was consistent with the 3-box motif being a structural feature of most BTB proteins of the CRL3 complex (12). Indeed, Btkα assembled within a classical CRL3 complex *in vivo*, which included Cul3 and Rbx1, and underwent Lys^48^ polyubiquitylation. This behavior was similar to that of other substrate receptors, representing a characteristic signature of cellular E3 feedback regulation mediated by autoubiquitylation (36).

Due to the occurrence of ankyrins, RCC1, and BTB domain within the same polypeptide (26), IBtkα is endowed with unique biochemical and functional properties as compared with other CRL3 substrate adaptors, because it could share multiple physical and functional interactions with members of cellular pathways. In the search of CRL3^IBTK^ substrates, we found that IBtkα *in vivo* associated with Pdcd4, a tumor suppressor involved in several cellular processes, including translation repression (37). Indeed, we proved that IBtkα promoted the ubiquitylation and subsequent proteasomal degradation of Pdcd4. In fact, the depletion of IBtkα by RNA interference caused the Pdcd4 accumulation without altering the *PDCD4* gene expression. Conversely, overexpression of IBtkα promoted the Pdcd4 degradation, and this effect was counteracted by the proteasome inhibitor MG132, indicating that the Pdcd4 ubiquitylation mediated by CRL3^IBTK^ was coupled to proteasomal degradation. Moreover, the mutant IBtkαΔBTB, which bound to Pdcd4 and not to Cul3, was unable to promote Pdcd4 ubiquitylation, suggesting the requirement of IBtkα interaction with Cul3 to promote the Pdcd4 ubiquitylation. Collectively, our results indicated that CRL3^IBTK^ targeted Pdcd4 for ubiquitylation coupled to proteasomal degradation.

Dorrello *et al.* (39) previously showed that in starved T98G glioblastoma cells, Pdcd4 was ubiquitylated/degraded following serum starvation/replenishment through the SCF^βTrcp^-dependent pathway, which required the phosphorylation of Pdcd4 at serines 67, 71, and 76. In the present study, we have found that CRL3^IBTK^ also promotes the Pdcd4 ubiquitylation/degradation upon serum starvation/replenishment. However, the IBtkα-dependent degradation of Pdcd4 does not require the presence of serines 67, 71, and 76 because the Pdcd4 S67A/S71A/S76A mutant, which is resistant to the SCF^βTrcp^-mediated degradation (39), still underwent to IBtkα-dependent degradation. These results indicate that the IBtkα-mediated degradation of Pdcd4 relies on regulatory mechanisms that differ from the SCF^βTrcp^ pathway. In this regard, proteins may be subjected to redundant ubiquitylation through multiple E3 ligases. For example, cyclin E1 is ubiquitylated by CRL3^RhoBTB3^ (46) or SCF^Fbx7^ during entry into S phase of the cell cycle (47). Thus, it is possible that different E3s cooperate in regulating the turnover of ligase targets whenever specific stimuli generate the appropriate signals to trigger the rapid ubiquitylation and degradation of substrates. Having identified CRL3^IBTK^ as adaptor of Pdcd4 for CRL3-dependent ubiquitylation, it will be relevant in the future to characterize the specific pathways activating this process.

Pdcd4 inhibits the translation of mRNAs with a structured 5′-UTR by repressing the eIF4A1 RNA helicase (40) as well as the assembly of distinct mRNAs, such as Bcl-xL, within the 48S initiation complex (42). Pdcd4 also inhibited the translation elongation through its binding to secondary structures of the coding region of mRNAs, such as A-*myb* and c-*myb* (38). Thus, we investigated whether IBtkα, promoting Pdcd4 degradation, could release the translation of mRNAs from Pdcd4 repression. By using two luciferase mRNA reporters, we observed that depletion of IBtkα by RNA interference inhibited the translation of mRNAs with stem-loop structured 5′-UTR and unstructured 5′-UTR, having a major effect on the stem-loop structured 5′-UTR. This evidence suggested that IBtkα promoted the Pdcd4-dependent mRNA translation by causing the degradation of the Pdcd4 repressor. IBtkα did not affect the global protein synthesis, as expected for a targeted action of IBtkα on Pdcd4-dependent translation. However, IBtkα promoted the translation of Bcl-xL mRNA, which indeed is subject to Pdcd4-translational repression (42). Thus, by targeting Pdcd4 for degradation, CRL3^IBTK^ could modulate the quality shift of mRNA translation under different cellular conditions. The endoplasmic reticulum stress up-regulates IBtkα at both transcriptional and translational levels as a likely mechanism of cell survival (48, 49). Moreover, a genome-wide RNAi screen identified *IBTK* as an essential function for viability of K-Ras mutant colorectal cancer cells, supporting a role of the *IBTK* gene in Ras-dependent pathways (50). Obtaining further insights into the regulation mechanisms of the CRL3^IBTK^ activity will probably clarify the specific role of IBtkα in different signaling pathways.

## Footnotes

5 The abbreviations used are:

Ub: ubiquitin
Cul: Cullin
CRL: Cul-RING ligase
SCF: Skp1/Cul1/F-Box
CRL3: Cullin3-RING ligase
BTB: Bric-a-brac, Tramtrack, and Broad Complex
POZ: pox virus and zinc finger
KLH: Kelch
RCC1: regulator of chromosome condensation 1
IP: immunoprecipitation
WB: Western Blot
RBD: RNA-binding domain
aa: amino acids
CHX: cycloheximide
RIPA: radioimmune precipitation assay.

## References

[1] Hershko A. Ciechanover A. (1998) The ubiquitin system. Annu. Rev. Biochem. 67, 425–479 9759494 10.1146/annurev.biochem.67.1.425

[2] Pickart C. M. (2004) Back to the future with ubiquitin. Cell 116, 181–190 14744430 10.1016/s0092-8674(03)01074-2

[3] Lipkowitz S. Weissman A. M. (2011) RINGs of good and evil: RING finger ubiquitin ligases at the crossroads of tumour suppression and oncogenesis. Nat. Rev. Cancer 11, 629–643 21863050 10.1038/nrc3120 PMC3542975

[4] Petroski M. D. Deshaies R. J. (2005) Function and regulation of cullin-RING ubiquitin ligases. Nat. Rev. Mol. Cell Biol. 6, 9–20 15688063 10.1038/nrm1547

[5] Saha A. Deshaies R. J. (2008) Multimodal activation of the ubiquitin ligase SCF by Nedd8 conjugation. Mol. Cell 32, 21–31 18851830 10.1016/j.molcel.2008.08.021 PMC2644375

[6] Guardavaccaro D. Pagano M. (2004) Oncogenic aberrations of cullin-dependent ubiquitin ligases. Oncogene 23, 2037–2049 15021891 10.1038/sj.onc.1207413

[7] Willems A. R. Schwab M. Tyers M. (2004) A hitchhiker's guide to the cullin ubiquitin ligases: SCF and its kin. Biochim. Biophys. Acta 1695, 133–170 15571813 10.1016/j.bbamcr.2004.09.027

[8] Cardozo T. Pagano M. (2004) The SCF ubiquitin ligase: insights into a molecular machine. Nat. Rev. Mol. Cell Biol. 5, 739–751 15340381 10.1038/nrm1471

[9] Bardwell V. J. Treisman R. (1994) The POZ domain: a conserved protein-protein interaction motif. Genes Dev. 8, 1664–1677 7958847 10.1101/gad.8.14.1664

[10] Zollman S. Godt D. Privé G. G. Couderc J. L. Laski F. A. (1994) The BTB domain, found primarily in zinc finger proteins, defines an evolutionarily conserved family that includes several developmentally regulated genes in *Drosophila*. Proc. Natl. Acad. Sci. U.S.A. 91, 10717–10721 7938017 10.1073/pnas.91.22.10717 PMC45093

[11] Pintard L. Willis J. H. Willems A. Johnson J. L. Srayko M. Kurz T. Glaser S. Mains P. E. Tyers M. Bowerman B. Peter M. (2003) The BTB protein MEL-26 is a substrate-specific adaptor of the CUL-3 ubiquitin-ligase. Nature 425, 311–316 13679921 10.1038/nature01959

[12] Genschik P. Sumara I. Lechner E. (2013) The emerging family of CULLIN3-RING ubiquitin ligases (CRL3s): cellular functions and disease implications. EMBO J. 32, 2307–2320 23912815 10.1038/emboj.2013.173 PMC3770339

[13] Furukawa M. He Y. J. Borchers C. Xiong Y. (2003) Targeting of protein ubiquitination by BTB-Cullin 3-Roc1 ubiquitin ligases. Nat. Cell Biol. 5, 1001–1007 14528312 10.1038/ncb1056

[14] Geyer R. Wee S. Anderson S. Yates J. Wolf D. A. (2003) BTB/POZ domain proteins are putative substrate adaptors for cullin 3 ubiquitin ligases. Mol. Cell 12, 783–790 14527422 10.1016/s1097-2765(03)00341-1

[15] Xu L. Wei Y. Reboul J. Vaglio P. Shin T. H. Vidal M. Elledge S. J. Harper J. W. (2003) BTB proteins are substrate-specific adaptors in an SCF-like modular ubiquitin ligase containing CUL-3. Nature 425, 316–321 13679922 10.1038/nature01985

[16] Stogios P. J. Downs G. S. Jauhal J. J. Nandra S. K. Privé G. G. (2005) Sequence and structural analysis of BTB domain proteins. Genome Biol. 6, R82 16207353 10.1186/gb-2005-6-10-r82 PMC1257465

[17] Zhuang M. Calabrese M. F. Liu J. Waddell M. B. Nourse A. Hammel M. Miller D. J. Walden H. Duda D. M. Seyedin S. N. Hoggard T. Harper J. W. White K. P. Schulman B. A. (2009) Structures of SPOP-substrate complexes: insights into molecular architectures of BTB-Cul3 ubiquitin ligases. Mol. Cell 36, 39–50 19818708 10.1016/j.molcel.2009.09.022 PMC2847577

[18] Zhang D. D. Lo S. C. Sun Z. Habib G. M. Lieberman M. W. Hannink M. (2005) Ubiquitination of Keap1, a BTB-Kelch substrate adaptor protein for Cul3, targets Keap1 for degradation by a proteasome-independent pathway. J. Biol. Chem. 280, 30091–30099 15983046 10.1074/jbc.M501279200

[19] Cullinan S. B. Gordan J. D. Jin J. Harper J. W. Diehl J. A. (2004) The Keap1-BTB protein is an adaptor that bridges Nrf2 to a Cul3-based E3 ligase: oxidative stress sensing by a Cul3-Keap1 ligase. Mol. Cell Biol. 24, 8477–8486 15367669 10.1128/MCB.24.19.8477-8486.2004 PMC516753

[20] Furukawa M. Xiong Y. (2005) BTB protein Keap1 targets antioxidant transcription factor Nrf2 for ubiquitination by the Cullin 3-Roc1 ligase. Mol. Cell Biol. 25, 162–171 15601839 10.1128/MCB.25.1.162-171.2005 PMC538799

[21] Kobayashi A. Kang M. I. Okawa H. Ohtsuji M. Zenke Y. Chiba T. Igarashi K. Yamamoto M. (2004) Oxidative stress sensor Keap1 functions as an adaptor for Cul3-based E3 ligase to regulate proteasomal degradation of Nrf2. Mol. Cell Biol. 24, 7130–7139 15282312 10.1128/MCB.24.16.7130-7139.2004 PMC479737

[22] Bordeleau M. E. Robert F. Gerard B. Lindqvist L. Chen S. M. Wendel H. G. Brem B. Greger H. Lowe S. W. Porco J. A. Jr. Pelletier J. (2008) Therapeutic suppression of translation initiation modulates chemosensitivity in a mouse lymphoma model. J. Clin. Invest. 118, 2651–2660 18551192 10.1172/JCI34753 PMC2423864

[23] Lindqvist L. Oberer M. Reibarkh M. Cencic R. Bordeleau M. E. Vogt E. Marintchev A. Tanaka J. Fagotto F. Altmann M. Wagner G. Pelletier J. (2008) Selective pharmacological targeting of a DEAD box RNA helicase. PLoS One 3, e1583 18270573 10.1371/journal.pone.0001583 PMC2216682

[24] Ohta A. Schumacher F. R. Mehellou Y. Johnson C. Knebel A. Macartney T. J. Wood N. T. Alessi D. R. Kurz T. (2013) The CUL3-KLHL3 E3 ligase complex mutated in Gordon's hypertension syndrome interacts with and ubiquitylates WNK isoforms: disease-causing mutations in KLHL3 and WNK4 disrupt interaction. Biochem. J. 451, 111–122 23387299 10.1042/BJ20121903 PMC3632089

[25] Liu W. Quinto I. Chen X. Palmieri C. Rabin R. L. Schwartz O. M. Nelson D. L. Scala G. (2001) Direct inhibition of Bruton's tyrosine kinase by IBtk, a Btk-binding protein. Nat. Immunol. 2, 939–946 11577348 10.1038/ni1001-939

[26] Spatuzza C. Schiavone M. Di Salle E. Janda E. Sardiello M. Fiume G. Fierro O. Simonetta M. Argiriou N. Faraonio R. Capparelli R. Quinto I. Scala G. (2008) Physical and functional characterization of the genetic locus of IBtk, an inhibitor of Bruton's tyrosine kinase: evidence for three protein isoforms of IBtk. Nucleic Acids Res. 36, 4402–4416 18596081 10.1093/nar/gkn413 PMC2490745

[27] Fiume G. Rossi A. Di Salle E. Spatuzza C. Mallardo M. Scala G. Quinto I. (2009) Computational analysis and *in vivo* validation of a microRNA encoded by the IBTK gene, a regulator of B-lymphocytes differentiation and survival. Comput. Biol. Chem. 33, 434–439 19782003 10.1016/j.compbiolchem.2009.08.001

[28] Janda E. Palmieri C. Pisano A. Pontoriero M. Iaccino E. Falcone C. Fiume G. Gaspari M. Nevolo M. Di Salle E. Rossi A. De Laurentiis A. Greco A. Di Napoli D. Verheij E. Britti D. Lavecchia L. Quinto I. Scala G. (2011) Btk regulation in human and mouse B cells via protein kinase C phosphorylation of IBtkγ. Blood 117, 6520–6531 21482705 10.1182/blood-2010-09-308080

[29] Fiume G. Rossi A. de Laurentiis A. Falcone C. Pisano A. Vecchio E. Pontoriero M. Scala I. Scialdone A. Masci F. F. Mimmi S. Palmieri C. Scala G. Quinto I. (2013) Eukaryotic initiation factor 4H is under transcriptional control of p65/NF-κB. PLoS One 8, e66087 23776612 10.1371/journal.pone.0066087 PMC3679033

[30] de Laurentiis A. Gaspari M. Palmieri C. Falcone C. Iaccino E. Fiume G. Massa O. Masullo M. Tuccillo F. M. Roveda L. Prati U. Fierro O. Cozzolino I. Troncone G. Tassone P. Scala G. Quinto I. (2011) Mass spectrometry-based identification of the tumor antigen UN1 as the transmembrane CD43 sialoglycoprotein. Mol. Cell. Proteomics 10.1074/mcp.M111.007898 PMC3098598 21372249

[31] de Laurentiis A. Caterino M. Orrù S. Ruoppolo M. Tuccillo F. Masullo M. Quinto I. Scala G. Pucci P. Palmieri C. Tassone P. Salvatore F. Venuta S. (2006) Partial purification and MALDI-TOF MS analysis of UN1, a tumor antigen membrane glycoprotein. Int. J. Biol. Macromol. 39, 122–126 16580720 10.1016/j.ijbiomac.2006.02.020

[32] Shevchenko A. Tomas H. Havlis J. Olsen J. V. Mann M. (2006) In-gel digestion for mass spectrometric characterization of proteins and proteomes. Nat. Protoc. 1, 2856–2860 17406544 10.1038/nprot.2006.468

[33] Käll L. Canterbury J. D. Weston J. Noble W. S. MacCoss M. J. (2007) Semi-supervised learning for peptide identification from shotgun proteomics datasets. Nat. Methods 4, 923–925 17952086 10.1038/nmeth1113

[34] Kumar N. Wethkamp N. Waters L. C. Carr M. D. Klempnauer K. H. (2013) Tumor suppressor protein Pdcd4 interacts with Daxx and modulates the stability of Daxx and the Hipk2-dependent phosphorylation of p53 at serine 46. Oncogenesis 2, e37 23536002 10.1038/oncsis.2012.37 PMC3564021

[35] Kelley L. A. Sternberg M. J. (2009) Protein structure prediction on the Web: a case study using the Phyre server. Nat. Protoc. 4, 363–371 19247286 10.1038/nprot.2009.2

[36] Weissman A. M. Shabek N. Ciechanover A. (2011) The predator becomes the prey: regulating the ubiquitin system by ubiquitylation and degradation. Nat. Rev. Mol. Cell Biol. 12, 605–620 21860393 10.1038/nrm3173 PMC3545438

[37] Lankat-Buttgereit B. Göke R. (2009) The tumour suppressor Pdcd4: recent advances in the elucidation of function and regulation. Biol. Cell 101, 309–317 19356152 10.1042/BC20080191

[38] Biyanee A. Ohnheiser J. Singh P. Klempnauer K. H. (2015) A novel mechanism for the control of translation of specific mRNAs by tumor suppressor protein Pdcd4: inhibition of translation elongation. Oncogene 34, 1384–1392 24681950 10.1038/onc.2014.83

[39] Dorrello N. V. Peschiaroli A. Guardavaccaro D. Colburn N. H. Sherman N. E. Pagano M. (2006) S6K1- and βTRCP-mediated degradation of PDCD4 promotes protein translation and cell growth. Science 314, 467–471 17053147 10.1126/science.1130276

[40] Yang H. S. Jansen A. P. Komar A. A. Zheng X. Merrick W. C. Costes S. Lockett S. J. Sonenberg N. Colburn N. H. (2003) The transformation suppressor Pdcd4 is a novel eukaryotic translation initiation factor 4A binding protein that inhibits translation. Mol. Cell Biol. 23, 26–37 12482958 10.1128/MCB.23.1.26-37.2003 PMC140682

[41] Yang H. S. Cho M. H. Zakowicz H. Hegamyer G. Sonenberg N. Colburn N. H. (2004) A novel function of the MA-3 domains in transformation and translation suppressor Pdcd4 is essential for its binding to eukaryotic translation initiation factor 4A. Mol. Cell Biol. 24, 3894–3906 15082783 10.1128/MCB.24.9.3894-3906.2004 PMC387765

[42] Liwak U. Thakor N. Jordan L. E. Roy R. Lewis S. M. Pardo O. E. Seckl M. Holcik M. (2012) Tumor suppressor PDCD4 represses internal ribosome entry site-mediated translation of antiapoptotic proteins and is regulated by S6 kinase 2. Mol. Cell Biol. 32, 1818–1829 22431522 10.1128/MCB.06317-11 PMC3347423

[43] Bennett E. J. Rush J. Gygi S. P. Harper J. W. (2010) Dynamics of cullin-RING ubiquitin ligase network revealed by systematic quantitative proteomics. Cell 143, 951–965 21145461 10.1016/j.cell.2010.11.017 PMC3008586

[44] Mathew R. Seiler M. P. Scanlon S. T. Mao A. P. Constantinides M. G. Bertozzi-Villa C. Singer J. D. Bendelac A. (2012) BTB-ZF factors recruit the E3 ligase cullin 3 to regulate lymphoid effector programs. Nature 491, 618–621 23086144 10.1038/nature11548 PMC3504649

[45] Jin L. Pahuja K. B. Wickliffe K. E. Gorur A. Baumgärtel C. Schekman R. Rape M. (2012) Ubiquitin-dependent regulation of COPII coat size and function. Nature 482, 495–500 22358839 10.1038/nature10822 PMC3292188

[46] Lu A. Pfeffer S. R. (2013) Golgi-associated RhoBTB3 targets cyclin E for ubiquitylation and promotes cell cycle progression. J. Cell Biol. 203, 233–250 24145166 10.1083/jcb.201305158 PMC3812982

[47] Koepp D. M. Schaefer L. K. Ye X. Keyomarsi K. Chu C. Harper J. W. Elledge S. J. (2001) Phosphorylation-dependent ubiquitination of cyclin E by the SCFFbw7 ubiquitin ligase. Science 294, 173–177 11533444 10.1126/science.1065203

[48] Thoreen C. C. Chantranupong L. Keys H. R. Wang T. Gray N. S. Sabatini D. M. (2012) A unifying model for mTORC1-mediated regulation of mRNA translation. Nature 485, 109–113 22552098 10.1038/nature11083 PMC3347774

[49] Baird T. D. Palam L. R. Fusakio M. E. Willy J. A. Davis C. M. McClintick J. N. Anthony T. G. Wek R. C. (2014) Selective mRNA translation during eIF2 phosphorylation induces expression of IBTKα. Mol. Biol. Cell 25, 1686–1697 24648495 10.1091/mbc.E14-02-0704 PMC4019499

[50] Luo J. Emanuele M. J. Li D. Creighton C. J. Schlabach M. R. Westbrook T. F. Wong K. K. Elledge S. J. (2009) A genome-wide RNAi screen identifies multiple synthetic lethal interactions with the Ras oncogene. Cell 137, 835–848 19490893 10.1016/j.cell.2009.05.006 PMC2768667

